# Supplementary material for: Multifaceted Assessment of Wastewater-Based Epidemiology for SARS-CoV-2 in Selected Urban Communities in Davao City, Philippines: A Pilot Study
Source: Int J Environ Res Public Health. 2022 Jul 19;19(14):8789. doi: 10.3390/ijerph19148789 (PMC9324557; doi:10.3390/ijerph19148789)
Supplement: Supplementary file 1 [file ijerph-19-08789-s001.zip › ijerph-1628742-supplementary.pdf]

Multifaceted Assessment of Wastewater-Based Epidemiology for SARS-CoV-2 in Selected Urban Communities in Davao City, Philippines: A Pilot Study

Supplementary Material

Table S1. General description of sampling sites.

Community	Flow Observations	Type of waterway	Water body receiving wastewater	General site description
23-C	slow flowing to flowing	Sewer pipe	Davao Gulf	Musty smell
76-A Bucana	stagnant to slow flowing, water level is dependent with the tides	Sewer pipe	Davao River	Brown color. Has black sediments. Foul smell.
Leon Garcia	flowing	Sewer pipe	Davao Gulf	Greasy, but not as greasy as the Monteverde site. Foul smell.
Matina Crossing	moderate to fast flowing (15.9 to 27.6 LPM)*	Sewer pipe	Matina River	Faint ammonia smell
Mintal	slow to moderate flow (0.95 to 3.6 LPM)*	Creek	Talomo River	No significant scent. Clear water.
Monteverde	stagnant to slow flowing	Creek	Davao Gulf	Strong, nauseating smell compared to all sites. Pungent smell of Ammonia. Looks greasy and quite turbid.

* Discharge in liters per minute (LPM) measured using YF-S201 G1/2 water flow meter sensor with range 1-30L/min.

Table S2. Summary of physico-chemical and hydrologic data per site of wastewater samples collected on November to December 2020.

**Barangay**	**Date** mm/date	**Time**	**Discharge Rate (LPM)**	**pH**	**ORP (mV)**	**Salinity (PSU)**	**Temp (°C)**	**TDS (mg/L)**
23-C	11/13	14:17	n.c.	7.24	-94.7	9.79	31.74	8415
	11/27	10:22	n.c.	7.22	-159.1	0.44	27.27	455
	12/04	12:08	n.c.	7.04	-1658.2*	0.52	28.14	528
	12/11	11:32	n.c.	6.99	-153.1	0.44	27.99	449
	Average			7.12	-135.63	2.80	28.79	2461.75
	Standard Deviation (SD)			0.126	83.522	4.662	2.006	3968.996
76-A Bucana	11/13	8:33	n.c.	7.04	-131.6	0.54	27.23	544
	11/23	8.15	n.c.	6.77	76.3	0.12	27.61	124
	11/30	9:02	n.c.	6.7	41.3	0.16	27.49	171
	12/07	14:28	n.c.	6.64	159.1	0.16	27.55	171
	Average			6.79	36.28	0.25	27.47	252.50
	Standard Deviation (SD)			0.177	122.332	0.198	0.167	195.592
Leon Garcia	11/13	11:51	n.c.	7.19	-152	0.67	28.18	678
	11/27	9:13	n.c.	7.25	-148.8	0.87	26.58	780
	12/04	11:19	n.c.	7.14	-141.2	0.74	27.56	743
	12/11	9:50	n.c.	7.17	-153.3	0.77	27.13	768
	Average			7.19	-148.83	0.76	27.36	742.25
	Standard Deviation (SD)			0.046	5.424	0.083	0.677	45.522
Matina Crossing	11/09	14:19	n.c.	7.27	-76.2	0.43	29.49	443
	11/23	10:56	27.60	7.22	-90	0.33	27.6	337
	11/30	11:30	26.50	6.94	-154.2	1	27.36	990

	12/07	11:55	15.90	6.99	-106.1	0.38	27.41	390
	Average			7.11	-106.63	0.54	27.97	540.00
	Standard Deviation (SD)			0.164	33.989	0.313	1.022	303.105
Mintal	11/09	15:44	n.c.	6.63	6.63	0.09	27.85	102
	11/23	13:24	2.45	7.33	-85.8	0.45	28.93	463
	11/30	13:33	0.95	7.31	-91.3	0.5	29.66	514
	12/07	15:40	3.60	7.17	125.9	0.43	29.41	437
	Average			7.11	-11.14	0.37	28.96	379.00
	Standard Deviation (SD)			0.328	101.809	0.187	0.801	187.416
Monteverde	11/13	11:13	n.c.	7.24	-245.6	1.41	28.05	1371
	11/27	8:40	n.c.	7.12	-217.1	3.81	27.18	3488
	12/04	10:53	n.c.	7.16	-155.8	0.9	27.56	891
	12/11	9:28	n.c.	7.29	-205.4	1.93	27.13	1839
	Average			7.20	-205.98	2.01	27.48	1897.25
	Standard Deviation (SD)			0.077	37.469	1.270	0.426	1128.916

*data cannot be verified and is not included in the discussion.
n.c. = data was not collected; LPM = liters per minute; ORP = Oxidation-Reduction Potential measured in millivolts (mV); PSU Practical Salinity Units; Temp =Temperature measured in Celsius; TDS = Total Dissolved Solids measured in milligrams per liter (mg/L).

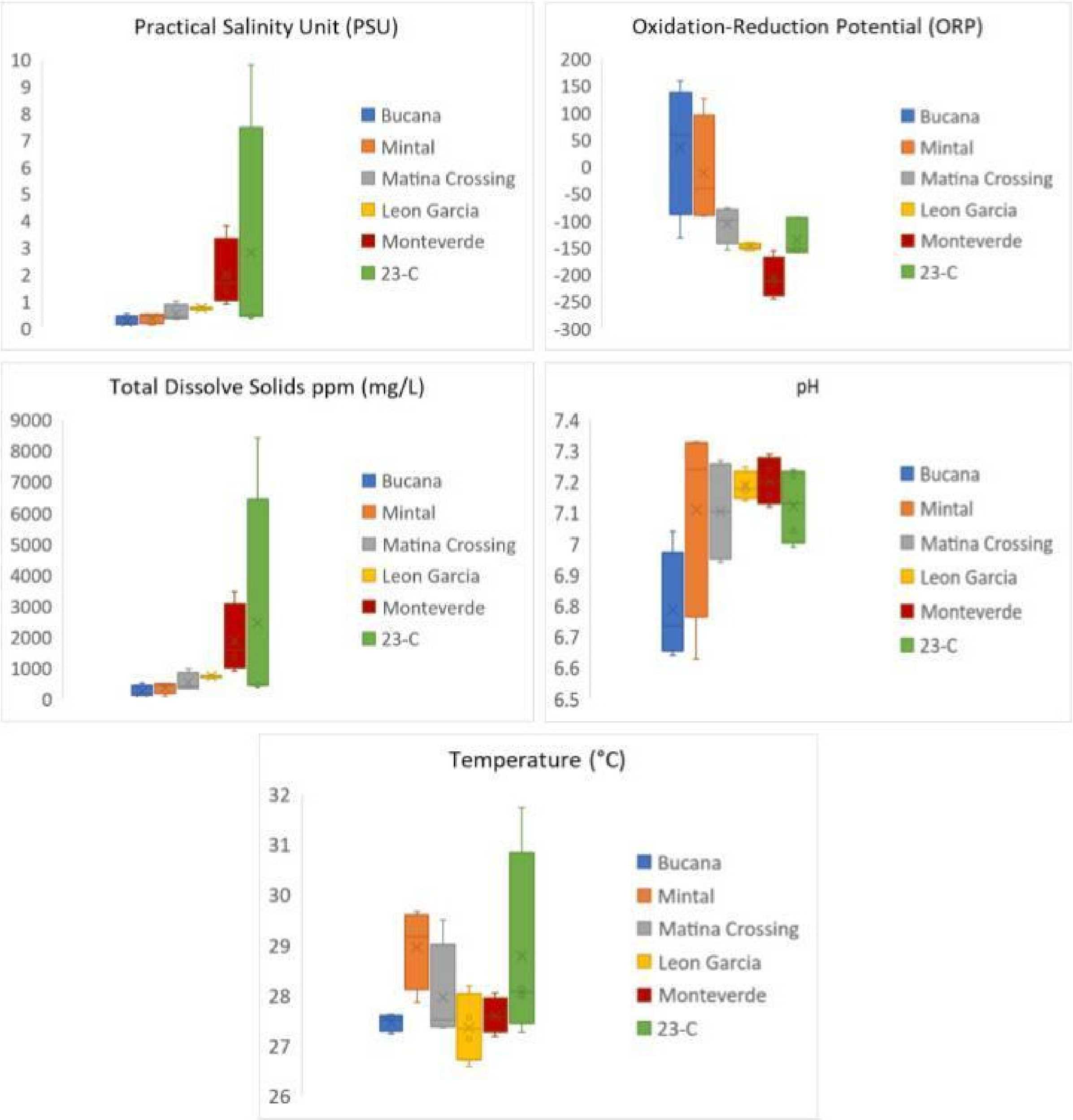

Figure S1. Boxplots of the physico-chemical attributes of each site during the 4 weeks of sample collection.

Table S3. Summary of Total and ***E. coli*** Coliform count.

**Barangay**	**Date** mo/date	**Time**	**Total Coliform (MPN/100mL)**	***E. coli*** **(MPN/100mL)**	**Total Coliform/ E. coli**	**E. coli / Total Coliform**
23-C	11/13	14:17	>2419000	>2419000	n.c.	n.c.
	11/27	10:22	>2419000	>2419000	n.c.	n.c.
	12/04	12:08	>2419000	>2419000	n.c.	n.c.
	12/11	11:32	>2419000	>2419000	n.c.	n.c.
76-A Bucana	11/13	8:33	>2419000	>2419000	n.c.	n.c.
	11/23	8.15	1,732,900	1,210,750	1.43	0.70
	11/30	9:02	>2419000	>2419000	n.c.	n.c.
	12/07	14:28	>2419000	>2419000	n.c.	n.c.
Leon Garcia	11/13	11:51	>2419000	>2419000	n.c.	n.c.
	11/27	9:13	>2419000	>2419000	n.c.	n.c.
	12/04	11:19	>2419000	>2419000	n.c.	n.c.
	12/11	9:50	>2419000	>2419000	n.c.	n.c.
Matina Crossing	11/09	14:19	>2419000	>2419000	n.c.	n.c.
	11/23	10:56	>2419000	>2419000	n.c.	n.c.
	11/30	11:30	>2419000	>2419000	n.c.	n.c.
	12/07	11:55	>2419000	>2419000	n.c.	n.c.
Mintal	11/09	15:44	853,700	120,500	7.08	0.14
	11/23	13:24	534,400	354,300	1.51	0.66
	11/30	13:33	923,450	114,650	8.05	0.12
	12/07	15:40	950,600	263,450	3.61	0.28
Monteverde	11/13	11:13	>2419000	>2419000	n.c.	n.c.
	11/27	8:40	1,732,900	1,236,950	1.40	0.71
	12/04	10:53	>2419000	>2419000	n.c.	n.c.
	12/11	9:28	>2419000	>2419000	n.c.	n.c.

n.c. cannot be computed

Table S4. Sampling, extraction, and SARS-CoV-2 detection data of wastewater samples from Davao City, Philippines.

Barangay	Date of collection	RNA Concentration (ng/uL)	CT mean values			Interpretation	Included in WGS (Yes/No)
			E gene	N gene	RdRP gene		
23-C	11/13/20	28.0	31.793	32.154	34.636	POSITIVE	Yes
	11/27/20	7.8	UND	UND	UND	NEGATIVE	No
	12/4/20	23.2	UND	38.739	35.283	POSITIVE	No
	12/11/20	35.8	UND	UND	38.597	POSITIVE	No
76-A Bucana	11/13/20	16.4	36.636	34.089	36.538	POSITIVE	No
	11/23/20	10.6	39.425	35.362	33.87	POSITIVE	Yes
	11/30/20	10.2	39.425	35.362	33.87	POSITIVE	No
	12/7/20	24.0	UND	38.266	UND	POSITIVE	No
Leon Garcia	11/13/20	27.2	31.69	31.257	33.484	POSITIVE	Yes
	11/27/20	19.4	37.088	35.591	35.008	POSITIVE	No
	12/4/20	40.2	39.727	31.702	31.893	POSITIVE	No
	12/11/20	36.6	32.037	29.406	31.264	POSITIVE	Yes
Matina Crossing	11/9/20	32.6	29.945	30.98	32.92	POSITIVE	Yes
	11/23/20	10.6	UND	35.771	32.572	POSITIVE	No
	11/30/20	22.8	35.55	32.002	32.19	POSITIVE	Yes
	12/7/20	21.2	33.09	34.124	33.225	POSITIVE	No
Mintal	11/9/20	22.6	UND	36.429	37.261	POSITIVE	No
	11/23/20	10.6	UND	UND	UND	NEGATIVE	No
	11/30/20	11.8	UND	UND	34.005	POSITIVE	No
	12/7/20	10.2	UND	35.457	35.997	POSITIVE	No

Monteverde	11/13/20	31.0	32.13	32.375	35.619	POSITIVE	Yes
	11/27/20	22.8	31.031	31.909	33.814	POSITIVE	Yes
	12/4/20	40.0	32.879	30.354	32.941	POSITIVE	Yes
	12/11/20	37.4	37.709	37.414	38.888	POSITIVE	No

Table S5. Regression analysis of N gene Ct prediction based on RNA concentration and Attack Rate.

	Coefficients	*95% CI*	*Standard Error*	*P-value*
Intercept	43.857	[39.450,48.264]	2.119	0.0000
RNA concentration	-0.201	[-0.355,-0.047]	0.074	0.0129
Attack Rate	-7.160	[-11.856,-2.464]	2.258	0.0046

Table S6. Regression analysis of E gene Ct prediction based on RNA concentration and Attack Rate.

	Coefficients	*95% CI*	*Standard Error*	*P-value*
Intercept	49.037	[44.401, 53.675]	2.229	0.0000
RNA concentration	-0.256	[-0.418, 0.094]	0.0779	0.0035
Attack Rate	-9.564	[-14.505, -4.623]	2.3758	0.0006

Table S7. Regression analysis of RdRP gene Ct prediction based on RNA concentration and Attack Rate.

	Coefficients	*95% CI*	*Standard Error*	*P-value*
Intercept	40.468	[36.140, 44.800]	2.082	0.0000
RNA concentration	-0.101	[-0.252, 0.050]	0.072	0.1796
Attack Rate	-4.902	[-9.520, -0.290]	2.218	0.0384

Table S8. Whole genome sequencing summary for selected wastewater samples from Davao City, Philippines.

Site (Date Collected)	Total No. of Ns	Genome Coverage (%)	Spike Gene Coverage (%)	Average read depth of genome coverage
23-C (11-13-20)	27,248	8.88	9.34	15.66
76-A Bucana (11-23-20)	24,457	18.21	18.08	20.74
Leon Garcia (11-13-20)	23,891	20.11	26.40	18.37
Leon Garcia (12-11-20)	23,957	19.88	17.74	15.23
Matina Crossing (11-09-20)	14,435	51.73	53.98	69.18
Matina Crossing (11-30-20)	29,561	1.14	0	0.43
Monteverde (11-13-20)	26,812	10.34	9.31	20.83
Monteverde (11-27-20)	22,568	24.53	11.46	29.34
Monteverde (12-04-20)	23,280	22.15	20.15	20.54

Table S9. Non-synonymous SNPs of SARS-CoV-2 detected in wastewater from Davao City, Philippines from November to December 2020.

SNP	Affected Gene	Sites	Reported Characteristics
1. P309S	ORF1a	Bucana, Matina Crossing	• Not yet reported
2. A1432V	ORF1a	Monteverde	• Reported in India [1]<br>• Implications and functional consequences not known
3. L1735S	ORF1a	Monteverde	• Not yet reported
4. T2016K	ORF1a	Matina Crossing	• Belongs to a low pathogenicity clade that surged in Singapore in early 2020 [2]<br>• Commonly detected in Asia [3]<br>• Occurs on the nucleic acid binding domain of NSP3 protein and potentially increases the nucleic acid binding affinity [4,5]
5. L3606-	ORF1a	Bucana, Matina Crossing	• Not yet reported
6. K3839R	ORF1a	Monteverde	• Not yet reported
7. L3930F	ORF1a	Leon Garcia	• Detected in a mild clinical case of COVID-19 in Pasay City, Philippines [6]

SNP	Affected Gene	Sites	Reported Characteristics
			• Correlated with increased Case Fatality Ratio (CFR) in Brazil [7]
8. A88V	ORF1b	Bucana, Matina Crossing	• Reported in India and the Philippines (Makati City) [5,8]<br>• Implications and functional consequences not known
9. P234L	ORF1b	Leon Garcia	• Reported in New York City, USA [9]<br>• Implications and functional consequences not known
10. A241S	ORF1b	Matina Crossing	• Not yet reported
11. A440V	ORF1b	Monteverde	• Not yet reported
12. V463-	ORF1b	Monteverde	• Not yet reported
13. D614G	S	Bucana, Matina Crossing, Monteverde, Leon Garcia	• High rate of global occurrence [3,10-12]]<br>• Increase in fitness and infectivity of the virus [10,12,13]<br>• Enhances virus binding efficiency to the human ACE2 receptor [10,13,14]<br>• Associated with higher viral loads that can enable the virus to infect much younger individuals [15,16]<br>• Introduces epitope changes that alter binding affinities of antibodies, although the mutation confers higher susceptibility to serum neutralization [13,17]
14. N39S	ORF6	Leon Garcia	• Not yet reported
15. putative Alternative Start Codon (AUG to GUG)	ORF7a	Leon Garcia	• Not yet reported
16. K2R	ORF7a	Leon Garcia	• Not yet reported
17. I3V	ORF7a	Leon Garcia	• Not yet reported
18. K32E	ORF7a	Leon Garcia	• Not yet reported
19. V21I	ORF7b	Leon Garcia	• Detected in a mild clinical case of COVID-19 in Pasay City, Philippines [6]
20. V117I	ORF8	Bucana	• Not yet reported
21. P13L	N	Bucana, Matina Crossing	• Commonly detected in Asia [3]<br>• Associated with decreased transmission and mortality due to a reduced binding affinity between the mutated N protein and M protein of the virus thus leading to a more inefficient process of virion assembly [18]
22. R203K	N	Monteverde	• High rate of global occurrence [3,11]

SNP	Affected Gene	Sites	Reported Characteristics
23. G204R	N	Monteverde	• Co-occurring mutations that confer a replication advantage over preceding variants (R203/G204) and increase virus fitness and virulence [12]<br>• May promote the binding of RNA by increasing the positive charge within the linker region to increase the RNP assembly efficiency, thereby accelerating virus replication [12]<br>• Associated with the increased transmission and virulence of B.1.1.7 (Alpha) and P.1 (Gamma) variants [12]
24. P383L	N	Monteverde	• Not yet reported
25. S183F	N	Monteverde	• Not yet reported
26. R185C	N	Monteverde	• Not yet reported
27. S197L	N	Monteverde	• Linked to mild outcome of COVID-19 [19]<br>• Located at the phosphorylated "RS-motif"necessary for the recruitment of host RNA helicase DDX1 which facilitates template readthrough and enables longer subgenomic mRNA synthesis [19]<br>• Predicted to increase the antigenicity value of the N protein and potentially affect its capability to elicit the production of antibodies [11,20]
28. A218V	N	Monteverde	• Not yet reported
29. T247I	N	Monteverde	• Unique mutation detected in cat isolates [21]

Table S10. Summary of SNP detection from wastewater samples.

**SNP**	**Affected Genes**	**Sites**							**Counts**
		**76-A Bucana**	**Matina Crossing**	**Monteverde**			**Leon Garcia**		
		11/23/20	11/9/20	11/13/20	11/27/20	12/4/20	11/13/20	12/11/20	
P309S	ORF1a	1	1						2
A1432V					1				1
L1735S						1			1
T2016K			1						1
L3606-		1	1						2
K3839R					1				1
L3930F							1		1
A88V	ORF1b	1	1						2
P234L							1		1
A241S			1						1
A440V				1					1
V463-				1					1
D614G	S	1	1	1	1	1	1	1	7
N39S	ORF6							1	1
Putative alternative start codon (AUG to GUG)	ORF7a							1	1
K2R								1	1
I3V								1	1
K32E								1	1
V21I	ORF7b							1	1
V117I	ORF8	1							1
P13L	N	1	1						2
R203K				1	1				2

G204R				1	1				2
P383L				1					1
S183F					1				1
R185C					1				1
S197L					1				1
A218V					1				1
T247I					1				1
**Total # SNPs**		6	7	6	10	2	3	7	41

Table S11. SARS-CoV-2 wastewater non-synonymous SNPs in the GISAID database of clinical cases from the Philippines in March 2020 to July 2021.

SNPs	Lineage	No. of Sequences	Collection Date (Month/Year)	Location
**Pre-Sampling Period**				
1. D614G	B.1.1	1	Jun 2020	Laguna, Philippines
	B.1.1.63	1	Jul 2020	Manila, Philippines
2. P13L	B.6	13	Mar, Apr, Jun 2020	Pasig, Philippines<br>La Union, Philippines<br>Manila, Philippines<br>Muntinlupa, Philippines
3. R203K/G204R	B.1.1	1	Jun 2020	Laguna, Philippines
	B.1.1.63	1	Jul 2020	Manila, Philippines
**Post-Sampling Period**				
1. D614G	AY.1	2	Aug 2021	Davao, Philippines
	AY.14	26	Jun, Jul, Aug 2021	Davao, Philippines
	AY.44	1	Jul 2021	Davao, Philippines
	AY.48	2	Jul 2021	Davao, Philippines
	AY.56	1	Aug 2021	Davao, Philippines
	AY.61	11	Jul, Aug 2021	Davao, Philippines
	AY.75	27	Jul, Aug 2021	Davao, Philippines
	AY.75.2	26	Jul, Aug 2021	Davao, Philippines
	AY.76	7	Jul, Aug 2021	Davao, Philippines
	AY.82	1	Aug 2021	Davao, Philippines

	AY.99	1	Apr 2021	Davao, Philippines
	AY.102	3	Aug 2021	Davao, Philippines
	AY.106	2	Jul 2021	Davao, Philippines
	AY.122	16	Jul, Aug 2021	Davao, Philippines
	AY.125	1	Aug 2021	Davao, Philippines
	AY.127	1	Aug 2021	Davao, Philippines
	B.1.1.7	74	Apr, May, Jun, Jul, Aug 2021	Davao, Philippines
	B.1.1.28	40	Jan, Feb, Apr, May, Jun, Jul 2021	Davao, Philippines
	B.1.1.184	5	Jan 2021	Davao, Philippines
	B.1.351	195	Mar, Apr, May, Jun, Jul, Aug 2021	Davao, Philippines
	P.3	10	Feb, Apr, Jun, Jul 2021	Davao, Philippines
	No Lineage	4	Jan, Jun, Jul 2021	Davao, Philippines
2. P13L	AY.99	1	Apr 2021	Davao, Philippines
	B.1.1.28	14	Jan, Feb, Apr, May, Jun 2021	Davao, Philippines
	B.6	1	Mar 2021	Davao, Philippines
3. R203K/G204R	AY.99	1	Apr 2021	Davao, Philippines
	B.1.1.7	71	Apr, May, Jun, Jul, Aug 2021	Davao, Philippines
	B.1.1.28	38	Jan, Feb, Apr, May, Jun, Jul 2021	Davao, Philippines
	B.1.1.63	1	Mar 2021	Davao, Philippines
	B.1.1.184	5	Jan 2021	Davao, Philippines

	B.1.351	1	Jul 2021	Davao, Philippines
	P.3	10	Jan, Feb, Apr, Jun, Jul 2021	Davao, Philippines
4. V21I	B.1.1.28	2	Jan, Feb 2021	Davao, Philippines
5. P383L	B.1.1.28	12	Jan, Feb, Apr 2021	Davao, Philippines
	B.1.1.184	1	Jan 2021	Davao, Philippines

References

1. Das, J.K.; Sengupta, A.; Choudhury, P.P.; Roy, S. Characterizing Genomic Variants and Mutations in SARS-CoV-2 Proteins from Indian Isolates. *Gene reports* **2021**, 101044.
2. Koyama, T.; Parida, L. Identification of a Low Pathogenicity Clade of SARS-CoV-2. **2020**.
3. Omotoso, O.E.; Olugbami, J.O.; Gbadegesin, M.A. Assessment of Intercontinents Mutation Hotspots and Conserved Domains within SARS-CoV-2 Genome. *Infect. Genet. Evol.* **2021**, *96*, 105097.
4. Muttineni, R.; Kammili, N.; Bingi, T.C.; Rao M, R.; Putty, K.; Dholaniya, P.S.; Puli, R.K.; Pakalapati, S.; Doodipala, M.R.; Upadhyay, A.A. Clinical and Whole Genome Characterization of SARS-CoV-2 in India. *PLoS One* **2021**, *16*, e0246173.
5. Tablizo, F.A.; Lapid, C.M.; Maralit, B.A.; Yap, J.M.C.; Destura, R. V; Alejandria, M.A.; Petronio-Santos, J.A.; Morado, E.K.D.; Dizon, J.G.A.; Llames, J.-H.S.; et al. Analysis of Sars-Cov-2 Genome Sequences From the Philippines: Genetic Surveillance and Transmission Dynamics. *medRxiv* **2020**, 1–30, doi:10.1101/2020.08.22.20180034.
6. Velasco, J.M.; Chinnawirotpisan, P.; Joonlasak, K.; Manasatienkij, W.; Huang, A.; Valderama, M.T.; Diones, P.C.; Leonardia, S.; Timbol, M.L.; Navarro, F.C. Coding-Complete Genome Sequences of 23 SARS-CoV-2 Samples from the Philippines. *Microbiol. Resour. Announc.* **2020**, *9*, e01031-20.
7. Kamikubo, Y.; Takahashi, A. Paradoxical Dynamics of SARS-CoV-2 by Herd Immunity and Antibody-Dependent Enhancement. **2020**.
8. Maitra, A.; Sarkar, M.C.; Raheja, H.; Biswas, N.K.; Chakraborti, S.; Singh, A.K.; Ghosh, S.; Sarkar, S.; Patra, S.; Mondal, R.K. Mutations in SARS-CoV-2 Viral RNA Identified in Eastern India: Possible Implications for the Ongoing Outbreak in India and Impact on Viral Structure and Host Susceptibility. *J. Biosci.* **2020**, *45*, 1–18.
9. Duerr, R.; Dimartino, D.; Marier, C.; Zappile, P.; Wang, G.; Lighter, J.; Elbel, B.; Troxel, A.B.; Heguy, A. Dominance of Alpha and Iota Variants in SARS-CoV-2 Vaccine Breakthrough Infections in New York City. *J. Clin. Invest.* **2021**, *131*.
10. Li, Q.; Wu, J.; Nie, J.; Zhang, L.; Hao, H.; Liu, S.; Zhao, C.; Zhang, Q.; Liu, H.; Nie, L. The Impact of Mutations in SARS-CoV-2 Spike on Viral Infectivity and Antigenicity. *Cell* **2020**, *182*, 1284–1294.
11. Salpini, R.; Alkhatib, M.; Costa, G.; Piermatteo, L.; Ambrosio, F.A.; Di Maio, V.C.; Scutari, R.; Duca, L.; Berno, G.; Fabeni, L. Key Genetic Elements, Single and in Clusters, Underlying Geographically Dependent SARS-CoV-2 Genetic Adaptation and Their Impact on Binding Affinity for Drugs and Immune Control. *J. Antimicrob. Chemother.* **2021**, *76*, 396–412.
12. Wu, H.; Xing, N.; Meng, K.; Fu, B.; Xue, W.; Dong, P.; Tang, W.; Xiao, Y.; Liu, G.; Luo, H. Nucleocapsid Mutations R203K/G204R Increase the Infectivity, Fitness, and Virulence of SARS-CoV-2. *Cell Host Microbe* **2021**, *29*, 1788–1801.
13. Plante, J.A.; Liu, Y.; Liu, J.; Xia, H.; Johnson, B.A.; Lokugamage, K.G.; Zhang, X.; Muruato, A.E.; Zou, J.; Fontes-Garfias, C.R. Spike Mutation D614G Alters SARS-CoV-2 Fitness. *Nature* **2021**, *592*, 116–121.
14. Zhang, L.; Jackson, C.B.; Mou, H.; Ojha, A.; Rangarajan, E.S.; Izard, T.; Farzan, M.; Choe, H. The D614G Mutation in the SARS-CoV-2 Spike Protein Reduces S1 Shedding and Increases Infectivity. *BioRxiv* **2020**.
15. Volz, E.; Hill, V.; McCrone, J.T.; Price, A.; Jorgensen, D.; O'Toole, Á.; Southgate, J.; Johnson, R.; Jackson, B.; Nascimento, F.F. Evaluating the Effects of SARS-CoV-2 Spike Mutation D614G on Transmissibility and Pathogenicity. *Cell* **2021**, *184*, 64–75.
16. Wang, R.; Hozumi, Y.; Yin, C.; Wei, G.W. Decoding SARS-CoV-2 Transmission and Evolution and Ramifications for COVID-19 Diagnosis, Vaccine, and Medicine. *J. Chem. Inf. Model.* **2020**, *60*, 5853–5865, doi:10.1021/acs.jcim.0c00501.
17. Koyama, T.; Weeraratne, D.; Snowdon, J.L.; Parida, L. Emergence of Drift Variants That May Affect COVID-19 Vaccine Development and Antibody Treatment. *Pathogens* **2020**, *9*, 324.
18. Oulas, A.; Zanti, M.; Tomazou, M.; Zachariou, M.; Minadakis, G.; Bourdakou, M.M.; Pavlidis, P.; Spyrou, G.M. Generalized Linear Models Provide a Measure of Virulence for Specific Mutations in SARS-CoV-2 Strains. *PLoS One* **2021**, *16*, e0238665.
19. Nagy, Á.; Pongor, S.; Győrffy, B. Different Mutations in SARS-CoV-2 Associate with Severe and Mild Outcome. *Int. J. Antimicrob. Agents* **2021**, *57*, 106272.
20. Can, H.; Köseoğlu, A.E.; Alak, S.E.; Güvendi, M.; Döşkaya, M.; Karakavuk, M.; Gürüz, A.Y.; Ün, C. Analysis of the Full Genome Sequences of SARS-CoV-2 Isolates to Determine Antigenic Proteins and Epitopes to Be Used for the Development of a Vaccine or a Diagnostic Approach for COVID-19. **2020**.
21. Elaswad, A.; Fawzy, M.; Basiouni, S.; Shehata, A.A. Mutational Spectra of SARS-CoV-2 Isolated from Animals. *PeerJ* **2020**, *8*, e10609.
